# Dynamic Evaluation of Liver Volume and Function in Associating Liver Partition and Portal Vein Ligation for Staged Hepatectomy

**DOI:** 10.1007/s11605-017-3389-y

**Published:** 2017-03-10

**Authors:** Ernesto Sparrelid, Eduard Jonas, Antonios Tzortzakakis, Ulrika Dahlén, Gustav Murquist, Torkel Brismar, Rimma Axelsson, Bengt Isaksson

**Affiliations:** 1 Division of Surgery, Department for Clinical Science, Intervention and Technology (CLINTEC), Karolinska Institutet, Karolinska University Hospital, 141 86 Stockholm, Sweden; 2 Division of Radiology, Department for Clinical Science, Intervention and Technology (CLINTEC), Karolinska Institutet, Karolinska University Hospital, Stockholm, Sweden; 3 Department of Medical Physics, Karolinska University Hospital, Stockholm, Sweden

**Keywords:** Colorectal cancer, Liver metastases, Liver function tests, ALPPS

## Abstract

**Background:**

Despite a fast and potent growth of the future liver remnant (FLR), patients operated with associating liver partition and portal vein ligation for staged hepatectomy (ALPPS) are at risk of developing posthepatectomy liver failure. In this study, the relation between liver volume and function in ALPPS was studied using a multimodal assessment.

**Methods:**

Nine patients with colorectal liver metastases treated with neoadjuvant chemotherapy and operated with ALPPS were studied with hepatobiliary scintigraphy, computed tomography, indocyanine green clearance test, and serum liver function tests. A comparison between liver volume and function was conducted.

**Results:**

The preoperative FLR volume of 19.5% underestimated the preoperative FLR function of 25.3% (*p* = 0.011). The increase in FLR volume exceeded the increase in function at day 6 after stage 1 (FLR volume increase 56.7% versus FLR function increase 28.2%, *p* = 0.021), meaning that the increase in function was 50% of the increase in volume. After stage 2, functional increase exceeded the volume increase, resulting in similar values 28 days after stage 2.

**Conclusions:**

In the inter-stage period of ALPPS, the high volume increase is not paralleled by a corresponding functional increase. This may in part explain the high morbidity and mortality rates associated with ALPPS. Functional assessment of the FLR is advised.

## Introduction

Indications for liver resection in colorectal liver metastases (CRLM) have changed over the last two decades, from the historical tumor-based criteria to the current paradigm where the focus is on the future liver remnant (FLR).^1^, ^2^ With the current widely accepted definition of resectability, resection is indicated when R0 resection of metastases, including extrahepatic disease, can be achieved while preserving a FLR sufficient for maintaining postoperative function and allowing adequate regeneration to restore sustainable liver function.^3^ There is increased focus on extending potential curative surgery to patients currently not eligible for treatment. Conversion strategies can be divided into tumor-directed treatments (multi-modality local tumor therapy and chemotherapy) or enhancement of postoperative hepatic functional reserve by manipulation of the FLR.^4^ Portal vein occlusion (PVO), by either portal vein embolization (PVE) or portal vein ligation (PVL), is a well-established technique for inducing hypertrophy of the FLR.^5^, ^6^ In a recently published meta-analysis, the two techniques (PVE and PVL) were found to be comparable in terms of growth induced and operative morbidity and mortality.^7^ Disease progression during the long waiting times leading to unresectable situations has been raised as a concern.^5^, ^8^–^10^

Associating liver partition and portal vein ligation for staged hepatectomy (ALPPS) is a novel two-stage technique intended to induce rapid growth of the FLR.^11^ Increase in FLR ranging from 65 to 110% and interval between the stage 1 and 2 procedures ranging from 6 to 15 days have been reported.^12^ Conceptually, ALPPS is potentially more versatile in its application. For example, the FLR is not confined to the classic sector-restricted FLR and a number of configurations with a single segment as FLR (so-called mono-segment ALPPS) have been described.^13^ Concern has been voiced that the extreme increase in size is the result of trauma-induced edema and swelling due to excessive portal flow, rather than a true hypertrophy.^14^ Furthermore, it is unclear whether the increase in volume due to hypertrophy translates into a comparable increase in function. Hepatobiliary scintigraphy (HBS) studies have shown that the increase in FLR function after PVE precede and is more pronounced than the increase in FLR volume.^15^ Recently, a case report and one preliminary report using HBS in ALPPS have suggested a discrepancy between liver volume and function, in that FLR volume instead overestimates FLR function after the stage 1 operation in ALPPS.^16^, ^17^

In this study, we investigate the dynamics of volume and function change in the FLR in patients operated with the ALPPS procedure after both stages of the operation, to explore whether increase in volume is translated into a corresponding increase in function.

## Materials and Methods

### Patients

In Stockholm County, all patients with colorectal liver metastases considered for intervention are discussed at a regional hepatobiliary multidisciplinary team conference for assessment and treatment planning. Patients with CRLM and response to neoadjuvant chemotherapy that could be rendered tumor free by an extended right-sided hemihepatectomy (segments IV-VIII), with or without local resection or ablation in the FLR, but where the FLR was insufficient (i.e., a future liver remnant to body weight—FLR/BW—of less than 0.5%) were eligible for the study. This was a prospective observational study that included an extensive repeated multimodal liver volume and function evaluation of each patient. Patients were included after obtaining informed consent. The nature and timing of study-related investigations are summarized in Table 1.

**Table 1:** Timing of study-related investigations

Time	4-phase CE-CT	HBS with SPECT/CT	ICG-C
Day 1 pre-stage 1	X	X	X
Day 1 post-stage 1	–	–	X
Day 6 post-stage 1	X	X	X
Day 1 post-stage 2	–	–	X
Day 7 post-stage 2	–	X	X
Day 28 post-stage 2	–	X	X

*CE-CT* contrast enhanced computed tomography, *HBS with SPECT/CT* hepatobiliary scintigraphy with SPECT/CT, *ICG-C* indocyanine green clearance test

### Operative Intervention and Postoperative Assessment

After mobilization of the right liver and cholecystectomy, the right portal vein was identified and divided using a surgical stapler (Endo GIA^™^ Universal with Tri-Staple^™^, Covidien, Dublin, Ireland). The right hepatic vein and pedicle containing the right hepatic artery and right bile duct were isolated and circled with rubber vessel loops. Any tumors in the FLR were resected or microwave ablated, and complete parenchymal transection to the inferior vena cava was performed immediately to the right of the falciform ligament using a cavitron ultrasonic surgical aspirator (CUSA®, Valleylab Inc, Boulder, CO, USA). The deportalized liver was wrapped in a plastic bag, a surgical drain was placed in the crevice created by the parenchymal division, and the abdomen was closed. If volumetry performed on the four-phase contrast enhanced computed tomography (CE-CT) on the sixth postoperative day showed sufficient hypertrophy of the FLR (resulting in a FLR/BW >0.5%), the stage 2 procedure was performed on day 7. At the stage 2 operation, the right pedicle (right hepatic artery and bile duct) and right hepatic vein were divided using surgical stapler and the deportalized liver was removed. The abdomen was closed retaining the surgical drain that was placed at the first operation.

### Indocyanine Green Clearance and Serum Liver Function Tests

Indocyanine green clearance (ICG-C) measured as plasma retention at 15 min, expressed as percentage (ICG-R15%), was performed using the LiMON® system (PULSION Medical System, Munich, Germany) after intravenous injection of 0.5 mg/kg of ICG dye (Verdye®, Diagnostic Green GmbH, Aschheim-Dornach, Germany). Prothrombin time measured as the international normalized ratio (INR) and serum bilirubin levels were measured daily from the day before stage 1 until day 7 after stage 2 and then again on day 28 after stage 2.

### CT Volumetry

For calculation of the total estimated liver volume (TELV), the standardized formula as proposed by Vauthey et al. was used.^18^ The FLR volume was measured on the preoperative day and day 6 after stage 1 on CE-CT and on days 7 and 28 after stage 2 on the CT from the SPECT/CT. The standardized FLR (sFLR) was calculated by dividing the FLR with TELV, and percentage FLR increase on day 6 after stage 1 and days 7 and 28 after stage 2 examinations was calculated with the preoperative TELV and preoperative FLR volume as references. The kinetic growth rate of the FLR volume was calculated separately for the three time intervals (between the stage 1 and 2 operations, for the first 7 days after stage 2 and for days 8–28 after stage 2) expressed as percentage change per day. All liver volume calculations were performed using Volume Viewer© (Voxtool 11) for AW Volume Share 5 implemented on an AW Workstation (GE Healthcare, Fairfield, CT, USA).

### Scintigraphy

**Fig. 1:**
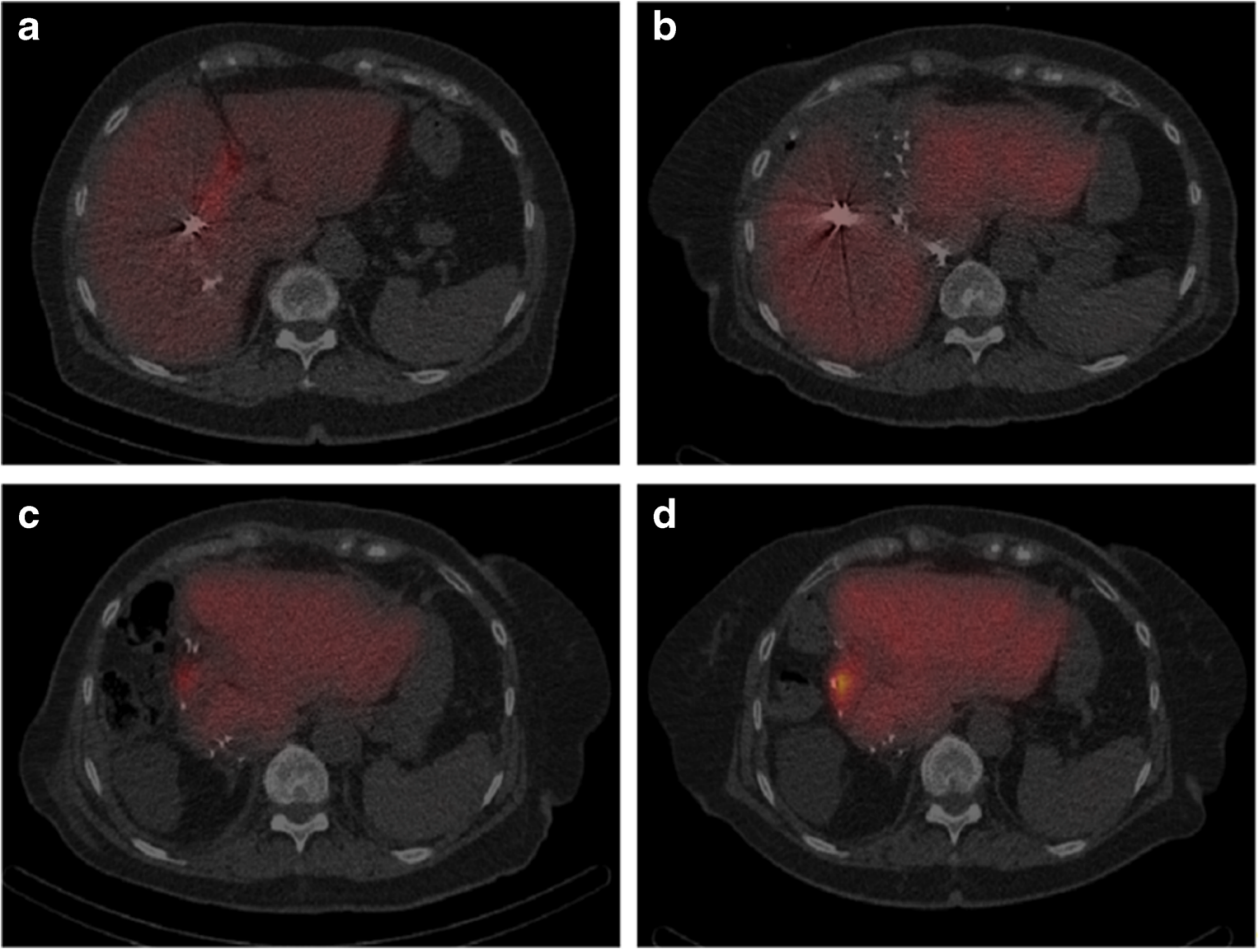
HBS with SPECT/CT in a patient with insufficient growth after previous PVE before stage 1 (**a**), on day 6 after stage 1 (**b**), day 7 (**c**), and day 28 (**d**) after stage 2

HBS and calculation of functional parameters were performed according to a method described previously.^19^, ^20^ Imaging was performed in the supine position using a large-field-of-view SPECT/CT camera (Symbia T-16, Siemens, Erlangen, Germany), equipped with low-energy high-resolution collimators, positioned over the liver and heart. Hepatic uptake function was calculated from dynamic acquisitions (36 frames of 10 s/frame, 128 matrix) performed directly after intravenous administration of 200 MBq ^99m^Tc-labeled (2,4,6 trimethyl-3-bromo) iminodiacetic acid (^99m^Tc-mebrofenin, Bridatec®, GE Healthcare, Milan, Italy). For three-dimensional assessment of liver function and calculation of functional liver volumes, the dynamic acquisition was followed by a fast SPECT acquisition (60 projections of 8 s/projection, 128 matrix) centered around the peak of the hepatic time-activity curve. Without repositioning the patient, a low-dose non-contrast-enhanced CT was performed for attenuation correction and anatomical mapping. Data were processed on a Hermes workstation (Hermes Medical Solutions AB, Stockholm, Sweden). An example of HBS with SPECT/CT at the four time points in a patient with insufficient growth after previous PVE is shown in Fig. 1.

### Calculations of Scintigraphic Functional Parameters

#### Planar Dynamic

Total liver ^99m^Tc-mebrofenin uptake rate (%/min), representing total liver function, was calculated from the dynamic acquisitions as geometric mean (Gmean = square root of the multiplication of anterior and posterior data sets) using the intensity values acquired 150 to 350 s after isotope injection from regions of interest (ROI) over the liver, the mediastinal blood pool (heart and large vessels), and the total field of view. To compensate for differences in individual metabolic requirements, total liver function was divided by body surface area according to the Mosteller formula and expressed as %/min/m^2^. For calculation of the FLR function, a ROI delineating the FLR was drawn. On the preoperative examination, the falciform ligament/umbilical fissure, as visible on anterior CT projections, was used to delineate the border between segments II/III and IV. On day 6 after stage 1 examination, the FLR was well demarcated by the crevice between segments II/III and segment IV facilitating drawing of ROIs in the FLR as well as the deportalized liver for calculation of deportalized liver function. Calculation of FLR function and deportalized liver function was done by dividing the added counts 150–350 s after isotope injection within the respective delineated ROIs by the total liver counts within the same time frame and multiplying this factor by the total liver ^99m^Tc-mebrofenin uptake rate with values expressed as %/min/m^2^. Mebrofenin uptake per liter of FLR tissue was calculated by dividing the FLR function (not corrected for body surface area) by FLR volume and expressed as %/min/l. Increase in FLR function on day 6 after stage 1 and on day 7 and day 28 after stage 2 was calculated with the preoperative FLR function as reference and expressed as percentage increase. Kinetic growth rate in FLR function was calculated for the three time intervals (between the stage 1 and 2 operations, during the first seven and days 8–28 after stage 2) by dividing the percentage increase for each time period with the number of elapsed days and expressed as percentage increase per day.

#### SPECT

For three-dimensional assessment of liver function, the liver was automatically delineated on the SPECT images by an outline extraction method (with a threshold of 30% of the maximal voxel count value). Activity within the bile ducts was eliminated by manual subtraction of extrahepatic bile duct activity and replacement of intrahepatic bile duct activity by an average density count of normal liver tissue. The FLR and deportalized liver (preoperatively the “to be deportalized liver”) were outlined manually on the SPECT/CT images with the CE-CT images used as reference. Total liver functional volume and FLR functional volume were subsequently calculated using the same threshold. The volume of the “to be deportalized liver functional volume” on the preoperative examination was estimated by subtracting the preoperative FLR functional volume from the preoperative total liver functional volume, whereas an actual calculation was performed for the day 6 deportalized liver functional volume, where the deportalized liver could clearly be delineated on the divided liver. The preoperative total liver functional volume was used as reference for calculation of both the preoperative and day 6 deportalized liver functional volume/total liver functional volume ratios.

### Data Collection and Statistical Analysis

Baseline patient characteristics, volumetric data, procedural data, and complications were collected prospectively in a local database. Median values with range or inter-quartile range were used for continuous variables whereas frequencies were calculated for categorical variables. The Wilcoxon signed-rank test was used to compare differences in liver volume and function, and the Spearman’s rank correlation coefficient was used to test for correlation between volumetric and functional parameters. Two-tailed *p* values of <0.05 were considered to represent statistical significance. Statistical analysis was performed using SPSS® Statistics, version 23 (IBM, Chicago, IL, USA) and GraphPad Prism®, version 7 (GraphPad Software, La Jolla, CA, USA).

The study was approved by the Regional Ethical Review Board (approval number 2013/353-31/1) and the Radiation Safety Committee at Karolinska University Hospital, Stockholm, Sweden.

## Results

### Patients

Between November 2012 and March 2014, nine patients with CRLM were included in the study. All patients received neoadjuvant chemotherapy consisting of FOLFOX or FOLFIRI with addition of a biological agent in two patients. A median of six cycles (range 4–14) of chemotherapy were given. All patients had response to this treatment according to RECIST criteria.^21^ In five patients, the liver metastases were detected synchronous to the primary tumor and two patients had tumor in the FLR. Six patients had previous failed PVO (PVE 3; PVL 2; PVL; and PVE 1) prior to inclusion, meaning that PVO did not induce sufficient growth of the FLR with a FLR/BW still below 0.5% after evaluation of the PVO effect. The stage 2 operation was performed in all patients 7 days after the stage 1 operation. Patient characteristics are summarized in Table 2.

**Table 2:** Patient characteristics before ALPPS

Variable	Patients (*n* = 9)
Median age, years	69 (41–77)
Male/female gender	6/3
Median BMI	26.1 (22.2–28.4)
ASA-class 1–2	5
ASA-class 3	4
Synchronous/metachronous metastases	5/4
Median number of liver metastases	4 (1–12)
Tumor localization	
Right lobe + segment 4	7
Right lobe + segment 4 + left lateral segment	2
Chemotherapy before ALPPS	
Oxaliplatin-based	5
Irinotecan-based	4
Targeted therapy	2
Number of chemotherapy cycles	6 (4–14)
Portal vein occlusion prior to ALPPS	
No portal vein occlusion	3
Portal vein embolization (PVE)	3
Portal vein ligation (PVL)	2
First PVL then PVE	1
FLR before PVO (ml)	219 (140–306)
sFLR before PVO (%)	13.6 (9.3–18.2)
FLR/BW before PVO (%)	0.30 (0.19–0.38)
FLR before ALPPS (ml)	300 (260–433)
sFLR before ALPPS (%)	19.5 (16.1–25.8)
FLR/BW before ALPPS (%)	0.41 (0.35–0.49)

Continuous variables are expressed as median with range in parentheses

*ASA* American society of anesthesiologists physical classification system, *TELV* total estimated liver volume, *FLR* future liver remnant, *sFLR* standardized FLR, *FLR/BW* FLR to body weight ratio

### Clinical Outcome

The median operating time for stage 1 was 280 min (range 200–498) and for stage two 47 min (29–107). Median intraoperative bleeding was 1500 ml (range 400–5600) for stage 1 and 150 ml (50–700) for stage 2. Two patients required tumor clearance in the FLR performed at the stage 1 operation, consisting of a local resection in segment 3 in one patient and a microwave ablation deep in the left lateral sector in the other. All patients completed the stage 2 operation with removal of segments IV-VIII. Pathological examination showed radical resection (R0) in seven patients while two patients had tumor cells within 1 mm from the resection line (R1). One patient with bile leakage after stage 2 required endoscopic stenting under general anesthesia, thus denoted as a grade 3b-complication according to the Clavien-Dindo classification.^22^ Three patients had pleural effusions requiring drainage under local anesthetic (grade 3a complication). No patient developed severe posthepatectomy liver failure and there was no 90-day mortality.

### Indocyanine Green Clearance and Serum Liver Function Tests

**Fig. 2:**
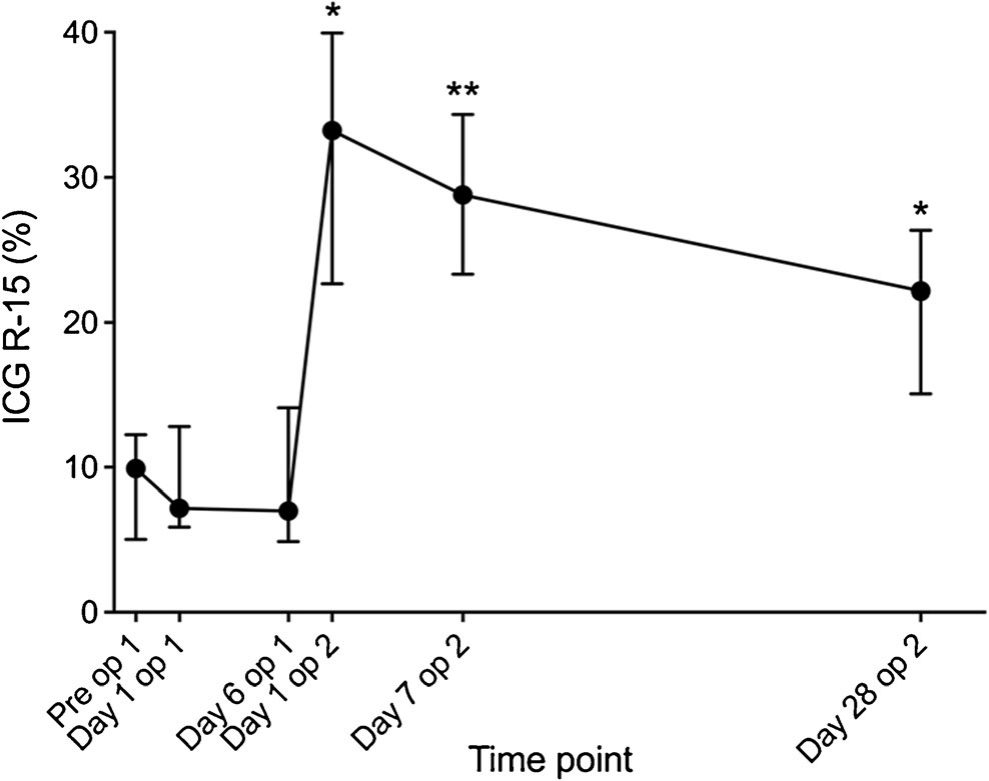
Indocyanine green retention at 15 min (ICG-R15%) at six time points before and after both stages of the ALPPS procedure. Data are presented as median with inter-quartile range. *p < 0.05, **p < 0.01

The median ICG-R15 on the day before stage 1 operation was 9.9% (range 1.2–20.7) and on day 6 after stage one 7.0% (4.2–19.5). There was a significant rise in ICG-R15 directly after stage 2 (33.3%, 8.5–43.2, *p* = 0.012) that was maintained at day 7 after stage 2 (28.8%, 19.2–38.9) and at day 28 after stage 2 (22.2%, 10.3–37.5). A graphical presentation of the ICG dynamics in ALPPS is shown in Fig. 2.

Median INR and bilirubin levels prior to the stage 1 operation were 1.0 (range 1.0–1.3) and 6 μmol/l (4–8), respectively. On day 5 after stage 1, median INR was elevated to 1.3 (range 1.1–1.5, *p* = 0.007) and bilirubin to 10 (7–22, *p* = 0.020), without any patients fulfilling the criteria for severe posthepatectomy liver failure according to the three most commonly used definitions.^23^–^25^ On day 5 after stage 2, both INR and bilirubin continued to rise with a median INR of 1.5 (range 1.2–1.8, *p* = 0.011) and bilirubin of 21 (12–49, *p* = 0.028), still without any patient developing severe posthepatectomy liver failure. There was a negative correlation between ICG-R15 levels at day 7 after stage 2 and the inter-stage increase in FLR volume (Spearman’s Rho = −0.68, *p* = 0.045), meaning that a high ICG-R15 level correlated with a lower increase in FLR volume.

### CT Volumetry

**Fig. 3:**
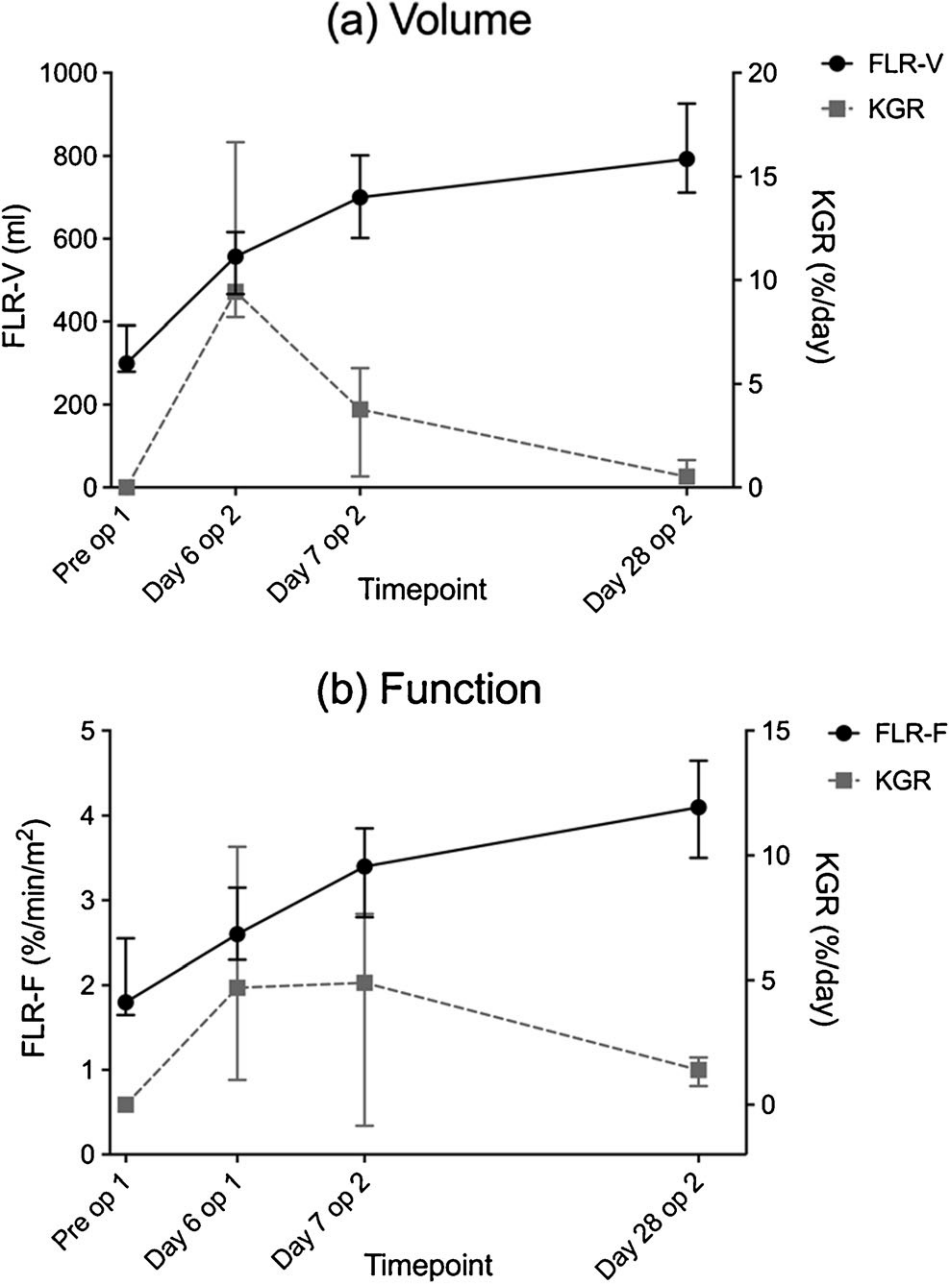
**a** FLR volume (FLR-V in ml) compared to kinetic growth rate (KGR) of volume increase (%/day) at the four time points: pre-stage 1, day 6 after stage 1, day 7 and 28 after stage 2. **b** FLR function (FLR-F as %/min/m^2^) compared to KGR of function increase (%/day) at the four time points: pre-stage 1, day 6 after stage 1, day 7 and 28 after stage 2. Data are presented as median with inter-quartile range

The median preoperative FLR volume was 300 ml (range 260–433) translating into a median sFLR of 19.5% (16.1–25.8). On day 6 after stage 1 and days 7 and 28 after stage 2, the FLR volume had increased to 557, 700, and 793 ml, respectively (*p* = 0.008, 0.011, and 0.008), translating into a median sFLR of 33.1, 40.6, and 48.0%. The median FLR volume percentage increase on day 6 after stage 1, day 7 and day 28 after stage 2 were 56.7% (range 32.3–110.4), 114.7% (48.8–174.3), and 132% (90–218.3) using the preoperative FLR volume and TELV as references. Median FLR/BW before stage 1 was 0.41% (range 0.35–0.49) and increased to 0.71% (0.54–0.90, *p* = 0.008) prior to stage 2. The median kinetic growth rate of the FLR volume was 9.4%/day (range 5.4–18.4) in the 6 days between stage 1 and 2, 3.8%/day (−0.3–8.2) the first 7 days after stage 2, and 0.5%/day (0.1–4.2) from day 8 to 28 after stage 2. The relation between increase in FLR volume and the kinetic growth rate of the FLR volume is demonstrated in Fig. 3a.

### Scintigraphic Functional Analysis

#### Planar Dynamic Parameters

The median preoperative FLR function was 1.8%/min/m^2^ (range 1.4–2.9), translating into a median FLR function/total liver function share of 25.3% (19.3–33.1). On day 6 after stage 1 and days 7 and 28 after stage 2, the median FLR function had increased to 2.6, 3.4, and 4.1%/min/m^2^, respectively (*p* = 0.051, 0.036, and 0.011), resulting in FLR function/total liver function shares of 33.9, 43.7, and 55.5%. Using the preoperative FLR function and total liver function share as reference, the median FLR function increase on day 6 after stage 1, day 7, and day 28 after stage 2 were 28.2% (range −35.7–83.8), 66.4% (0.7–147.5), and 92.2% (47.3–191.5), respectively. It was notable that the median FLR uptake rate per volume unit was decreased significantly on day 6 after stage 1 (8.5%/min/l) compared to preoperatively (11.8%/min/l, *p* = 0.028), and did not surpass the preoperative values on day 7 (9.0%/min/l) or on day 28 after stage 2 (10.1%/min/l). The median kinetic growth rate of the FLR function was 4.7%/day (range −6–14) in the 6 days between stage 1 and 2. During the 7 days following stage 2, it was 4.9%/day (range −2–24.9) and from day 8 to 28 after stage two 1.4%/day (−0.3–2.2). The relation between increase in FLR function and the kinetic growth rate of FLR function is demonstrated in Fig. 3b.

#### SPECT Parameters

An increase in the total liver functional volume (median values 1322 and 1637 ml, *p* = 0.008) was seen on day 6 after stage 1 whereas the “to be deportalized liver functional volume” and deportalized liver functional volume (941 and 1014 ml) did not increase significantly. A slight decrease in the deportalized liver function was seen prior to stage 2 as compared to the “to be deportalized liver function” prior to stage 1 (7.0%/min/m^2^ and 8.3%/min/m^2^), but it did not reach statistical significance (*p* = 0.208). There was no correlation between the increase in FLR function and decrease in deportalized liver function prior to stage 2 (Spearman’s Rho = −0.42, *p* = 0.265).

#### Volume Versus Function

**Fig. 4:**
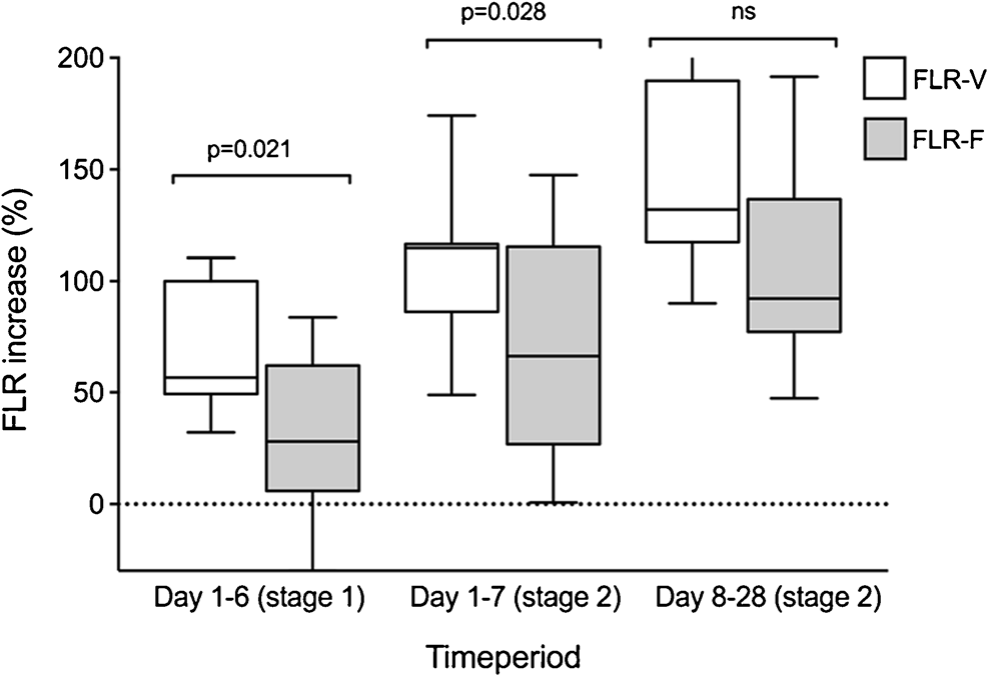
Comparison of percentage increase in FLR volume (FLR-V) and function (FLR-F) day 6 after stage 1 and days 7 and 28 after stage 2, with preoperative FLR-V and FLR-F as reference. Data are presented as median with range. *ns* not significant

The preoperative sFLR of 19.5% underestimated the preoperative FLR function/total liver function share of 25.3% (*p* = 0.011). The median increase in volume exceeded the increase in function at day 6 after stage 1 (FLR volume increase 56.7% versus FLR function increase 28.2%, *p* = 0.021). The increase in volume still exceeded increase in function the first 7 days after stage 2 (FLR volume increase 114.7% versus FLR function increase 66.4%, *p* = 0.028) but with greater functional growth rate after stage 2 resulted in comparable levels on day 28 (FLR volume increase 132.0% versus FLR function increase 92.2%, *p* = 0.11), with the preoperative FLR volume and FLR function as reference. The perioperative relation between FLR volume and FLR function increase is also demonstrated in Fig. 4.

**Fig. 5:**
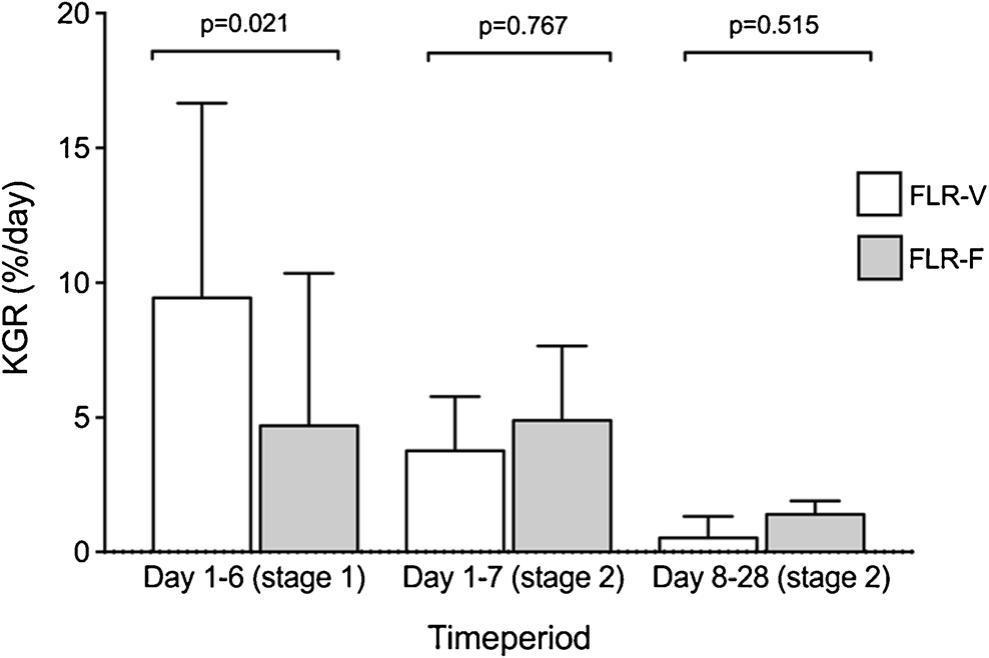
Median percentage change per day (KGR) in FLR volume (FLR-V) and function (FLR-F) between stage 1 and 2 operations, during the first 7 days after stage 2 and during days 8–28 after stage 2 as measured on CT volumetry (FLR-V) and planar dynamic scintigraphy (FLR-F). Data are presented as median with inter-quartile range

The median kinetic growth rate for FLR volume and FLR function between the stage 1 and 2 operations, during the first 7 days after stage 2 and days 8–28 after stage 2 measured on CT volumetry and planar dynamic scintigraphy, is shown in Fig. 5.

## Discussion

The ALPPS procedure has been suggested as an alternative to PVO for inducing hypertrophy of the FLR.^11^ Proponents claim a quicker increase in FLR in a shorter time as compared to conventional methods for FLR manipulation.^12^ Resection rates for patients with CRLM after ALPPS of 97–100% have been reported, compared to around 70–80% after PVO.^12^ Tumor recurrence rates at 1-year follow-up for ALPPS and PVO were comparable.^26^ As mentioned previously, skeptics have raised concern that the extreme increase in FLR size is not necessarily the result of true hypertrophy.^14^ Furthermore, the relationship between hypertrophy and increased function has been questioned.^15^–^17^ Also of concern is the safety of the ALPPS procedure, with 90-day mortality rates of 15% having been reported, compared to 6% for surgery after PVO.^26^

Of the nine patients included in this case series, six had failed PVO before inclusion. In spite of that, the median increase of 56.7% in FLR volume in only 6 days exceeded the growth that can be expected after PVO (mean increase of 39% after a mean of 45 days for PVE; mean increase of 27% after a mean of 59 days for PVL).^7^ Interestingly though, the kinetic growth rate for volume exceeded the kinetic increase in function, measured as percentage increase per day. The 56.7% median increase in FLR volume was paired with a 28.2% FLR function increase day 6 after stage 1, or in other words the functional increase represented only 50% of the increase in volume. This was also reflected as a decreased FLR uptake rate per volume unit on day 6 (median 8.5%/min/l on day 6 after stage 1 compared to 11.8%/min/l preoperatively). This gives some legitimacy to fears that the fast initial growth in volume after ALPPS does not translate into an equivalent increase in function.^14^ This may, in part, explain the observation that extreme hypertrophy does not necessarily ensure a sufficient FLR and safe postoperative course.^27^ However, a higher kinetic increase in FLR function than FLR volume after the stage 2 operation results in comparable values in FLR volume and FLR function on day 28 after stage 2.

In a letter to the editor, Lau et al. presented a case report suggesting using repeated ICG clearance measurement for resectability decision-making in ALPPS.^28^ In the present study, ICG-R15 values did not increase directly after stage 1 or prior to stage 2. The potent increase in ICG-R15 seen from day 1 after stage 2 did not translate into liver failure, and might simply be a result of increased blood flow to the liver remnant after resecting the deportalized liver. Whether a sharp increase in ICG-R15 prior to stage 2 could indicate a need to postpone stage 2 operation remains to be investigated in larger studies.

This study has some limitations. One obvious limitation is the small number of patients included in the study. This is a common problem for most studies in the field of PVO and ALPPS due to the limited number of patients that are subjected to these procedures. Still, the main findings in the present study are supported by initial experiences from other recent reports in that the FLR volume seems to overestimate FLR function after stage 1 of the ALPPS procedure.^16^, ^17^ Another potential confounder is that a majority of the patients in this study (six out of nine) were subjected to rescue ALPPS (after failed PVO). One could speculate that the hypertrophy of the FLR after ALPPS in a patient with previous PVO might be less pronounced compared to ALPPS upfront. However, there is currently no evidence to support this hypothesis. In fact, there are indications that the growth of the FLR after rescue ALPPS is similar to ALPPS upfront.^29^, ^30^ Finally, several factors are considered to impair the growth of FLR. Among the most commonly described are pre-procedural chemotherapy, high bilirubin levels, concomitant cholangitis, and diabetes mellitus.^31^, ^32^ However, result from different studies are conflicting and the evidence grade is poor.^33^ Compared to many previous studies in ALPPS, the patients in this study represent a comparatively homogenous cohort in that they all received chemotherapy for CRLM only, no patient had high bilirubin, cholangitis, or diabetes, and they were all operated with an extended right-sided hemihepatectomy on the seventh day after stage 1 operation. Still, until more solid evidence is presented, interpretation of the results in the present study has to be undertaken with these limitations in mind.

## Conclusion

In conclusion, this study supports previous reports indicating that ALPPS result in superior increases in both volume and function of the FLR as compared to PVO. However, the dynamics in terms of growth differ, with the inter-stage increase in volume not being matched with an equivalent increase in function. This discrepancy is neutralized by a more rapid increase in function after the second operation. Caution is advised in proceeding to second-stage procedures solely on volume-based assessments, especially when volumetric assessment is performed less than a week after the initial operation. The addition of functional parameters will probably result in more prudent decision-making and could lower mortality and morbidity in patients subjected to this important, but potentially dangerous intervention.
